# Efficient Concentration of Functional Polyphenols Using Their Interaction with Gelatin

**DOI:** 10.3390/foods10040698

**Published:** 2021-03-25

**Authors:** Mizuki Hirai, Ryo Kobori, Ryo Doge, Issei Tsuji, Akiko Saito

**Affiliations:** Graduate School of Engineering, Osaka Electro-Communication University (OECU), 18-8 Hatsu-cho, Neyagawa-shi, Osaka 572-8530, Japan; de18a001@oecu.jp (M.H.); de19a001@oecu.jp (R.K.); de20a001@oecu.jp (R.D.); me19a008@oecu.jp (I.T.)

**Keywords:** polyphenol compound–gelatin interaction, flavan-3-ol derivatives, green tea polyphenols

## Abstract

Among polyphenol compounds, the flavan-3-ol structure, which is the basic unit of green tea catechins and the galloyl groups contained in green tea catechins are known to exhibit various functions. In this paper, we discuss how to concentrate highly functional polyphenol compounds by exploiting the interaction between gelatin and the catechol structures. First, we confirmed the interaction between heat-stabilized gelatin and flavan-3-ol derivatives, including synthesized compounds. When green tea leaf extract containing a large amount of flavan-3-ol derivatives was incubated with gelatin, most of the polyphenol compounds it contained were adsorbed. Because the compounds adsorbed on gelatin could not be eluted, DPPH radical and ABTS radical scavenging activity tests were conducted using the as-prepared gelatin–polyphenol complex. Radical scavenging activity was observed when the compounds were adsorbed on gelatin and heating at 90 °C for 5 min did not have a significant effect on their activity. These results suggest that functional polyphenols can be efficiently concentrated using heat-stabilized gelatin and retain their functionality while adsorbed.

## 1. Introduction

There is currently great interest in compounds from food sources with antioxidant and/or biological activity as they are generally considered highly safe owing to their consumption as part of the general daily diet. In particular, the polyphenols contained in many health foods, as well as vegetables and fruits, are thought to have various health benefits [1,2].

Flavonoids, the generic term for flavones, anthocyanins, flavonols, flavan-3-ols (flavanols) and chalcones, are thought to promote health because of their various functionalities. Flavonoids are usually present in plants as a mixture of numerous compounds, and thus, bioactivity measurements of plant extracts show a mixture of activities. To clarify the mechanisms of their functionality, it is necessary to examine each compound in detail. However, it is currently difficult to obtain each polyphenol compound present in trace amounts in plants. Therefore, we developed a stereoselective synthesis method for producing flavan-3-ol derivatives, oligomeric compounds [3,4,5,6,7,8], galloyled derivatives [9,10,11,12,13] and acylated derivatives [14,15,16] for SAR studies, such as anti-oxidative activity [5,6,7,8,9], radical scavenging activity [10,11], HeLa S3 proliferation-inhibitory activity [7,12,13,16] and anti-viral activity [17]. These studies showed that fine structural differences, such as stereochemistry and the presence or absence of modifications, are important for demonstrating biological activity. Based on these SAR studies, it was suggested that the more galloyl groups there are in the molecule and the more oligomeric the structure is, the higher the general functionality. Therefore, we aimed to develop a method to efficiently extract galloyl-containing and oligomer-structured compounds from plants.

Among the flavan-3-ol derivatives, those with an oligomer structure are called proanthocyanidins, or condensed tannin. Furthermore, flavan-3-ols and proanthocyanidins are often galloylated and are known to be highly functional polyphenols. Tannins are cocktails extracted from vegetables that are used in the tanning process of converting animal skin to leather, which relies on the polyphenol‑collagen protein interaction in the skin. In general, polyphenol compounds, including flavan-3-ol derivatives such as proanthocyanidins, are considered to have non-specific interactions with various proteins; these interactions are commonly considered to largely contribute to the poor bioavailability of polyphenol compounds [18,19]. On the other hand, the binding mode between polyphenols and collagen has been clarified and it is known that the catechol structure binds to the glycine and proline residues that are abundant in collagen molecules [20]. Because catechol is contained in the galloyl and flavan-3-ol structures, they are considered to have highly functional galloyl structures and flavan-3-ol oligomers (proanthocyanidins) can be efficiently extracted from plants by utilizing their interaction with collagen. In recent years, an increasing number of reports have shown that the activity and stability of polyphenols can be enhanced by interacting with a protein. According to Song et al., when gelatin (chemically treated collagen) and flavonoid compounds were mixed and their cytotoxicity against lung cancer cells A549 was measured, the activity was higher when bound to gelatin and the flavonoid compounds were stabilized, enabling storage for a long period [21].

Examining the relationship between the structure and binding of compounds that are not commercially available [22], such as whether they interact with collagen or gelatin, is very difficult. Therefore, we verified that these compounds can be adsorbed and immobilized on gelatin using flavan-3-ol derivatives that can be secured by organic synthesis. We used a group of compounds that exist or are thought to exist as trace components in nature. The adsorbent was heat-stabilized gelatin, which is insoluble in water at room temperature (normal gelatin is soluble in water). When water-soluble collagen is used, it interacts with polyphenols to form a precipitate, but this may allow the compounds to enter between the gelatin fibers. By using water-insoluble heat-stabilized gelatin at room temperature, it is possible to adsorb compounds with galloyl groups and flavan-3-ol derivatives on the surface of the gelatin particles and, thus, we proposed using it as-prepared. In this report, we describe an adsorption experiment on heat-stabilized gelatin using a group of synthesized compounds and a polyphenol compound adsorption experiment from green tea extract using heat-stabilized gelatin. As a result, compounds with galloyl groups and flavan-3-ol oligomers (proanthocyanidins) were strongly adsorbed on gelatin. Most galloyl-containing compounds and flavan-3-ol derivatives were efficiently extracted from green tea leaves using heat-stabilized gelatin and the radical scavenging activity could be measured in the adsorbed state.

## 2. Materials and Methods

### 2.1. Reagents and Materials

All commercially available chemicals were used without further purification. (+)-Catechin (**1**), (−)-epicatechin (**2**), (−)-epigallocatechin-3-*O*-gallate (**3**) and (+)-gallocatechin-3-*O*-gallate (**4**) were purchased from Sigma-Aldrich (St. Louis, MO, USA). Compounds **5** to **14** were synthesized and stored. These compounds and the extracts obtained from green tea leaves were dissolved in molecular-biology-grade dimethyl sulfoxide (DMSO) and stored at −40 °C until further use. HPLC-grade methanol used for the extraction of green tea leaves was obtained from FUJI FILM WAKO Pure Chemical (Osaka, Japan). LC-MS-grade acetonitrile and HPLC-grade formic acid were obtained from Honeywell International (Charlotte, NC, USA). The 1,1-diphenyl-2-picrylhydrazyl (DPPH) radical was obtained from Tokyo Chemical Industry (Tokyo, Japan). All other reagents and chemicals were special grade and purchased from FUJI FILM WAKO Pure Chemical (Osaka, Japan) unless otherwise stated. The gelatin used in this experiment was heat-stabilized by heating porcine gelatin at 150 °C for 6 h and was provided by Nippi (not commercially available, Tokyo, Japan). Raw green tea leaves (*Camellia sinensis*) purchased from Kyoto OBUBU Tea Farm (Kyoto, Japan) were used. The leaves collected on the morning of delivery were received the next day and stored at −80 °C in a nitrogen atmosphere until just before use. Absorbance and LC-MS analyses were performed using a UV mini 1240 and LC-MS-2020, respectively (Shimadzu, Kyoto, Japan).

### 2.2. Adsorption of Flavan-3-ol Derivatives on Heat-Stabilized Gelatin

Water (1.0 mL) was added to 10 mg of heat-stabilized gelatin powder and incubated for 12 h at 25 °C. Gelatin absorbs water and increases in volume and, thus, 0.5 mg of dry heat-stabilized gelatin powder corresponded to 200 μL of gelatin suspension. After removing the supernatant by centrifugation, the suspension was washed three times with water, acetone and water in that order. The gelatin suspension was stored in the refrigerator until just before use. Furthermore, before use, the suspension was washed with the solvent and water used in the experiment.

#### 2.2.1. Adsorption of Flavan-3-ol Derivatives on Heat-Stabilized Gelatin

To 100 μL of the gelatin suspension (equivalent to 0.25 g of dry gelatin powder), 125 μL of 10% aqueous ethanol and 1 μL of a DMSO solution of each compound (10 mM) were added, vortexed, stirred and incubated for 1 h at room temperature. Ethanol (10%) was used because some compounds may precipitate when added to 100% water. The gelatin and supernatant were separated by centrifugation at 15,000 rpm for 5 min and the absorbance of the supernatant was measured at 280 nm. The precipitated gelatin was then washed with water and a 70% aqueous acetone solution and the absorbance of the washing solutions at 280 nm was measured. The amount of each compound adsorbed on the gelatin was calculated from the concentrations of the measured eluted compounds.

#### 2.2.2. Adsorption of Green Tea Leaf Extract on Gelatin

The surfaces of the raw green tea leaves were lightly washed with water and then immersed in methanol to extract the polyphenol compounds. The residue was filtered off, methanol was concentrated and removed to give a polyphenol mixture and a 100 mg/mL DMSO solution was prepared. To 100 μL of the gelatin suspension 125 μL of 10% aqueous ethanol and 1 μL of the DMSO solution of the extract was added and the mixture was vortexed, stirred and incubated for 1 h at room temperature. The gelatin and supernatant were separated by centrifugation at 15,000 rpm for 5 min and the HPLC spectra of the supernatant were measured. The fraction eluted from the gelatin with methanol was also analyzed by HPLC.

### 2.3. LogP Measurement (Octanol/Water Partition Coefficient)

The octanol/water partition coefficient was measured using the flask shaking method [23]. After mixing octanol and water at a ratio of 1:1 and stirring vigorously, the mixture was allowed to stand for 24 h for equilibration. A 10 mM solution of each compound was added to the equilibrated octanol-water mixture and the mixture was shaken for 1 h. Centrifugation was performed at 2000× *g* to separate the octanol and the aqueous layers. The absorbance of each layer at 280 nm was measured and the concentration was calculated from the calibration curve of each compound. The concentration of each compound was converted into Log*P*_OW_ as follows: [compound concentration of octanol layer/compound concentration of water layer.]

### 2.4. HPLC-MS Analysis Conditions

HPLC-MS analysis was performed using an LCMS-2020 system equipped with a DGU-20A degas unit, LC-20A binary pump, SIL-20AC autosampler, SPD-M20A diode array detector, CTO-20AC column oven and CBM-20A communications bus module connected to an LC workstation (Shimadzu, Kyoto, Japan). A COSOMOSIL Packed Column C18 (φ 150 mm × 4.6 mm, 5 μm, NACALAI TESQUE, Kyoto, Japan) was selected. Briefly, mobile phase A was a 400:10:1 mixed solvent of water, acetonitrile and formic acid and mobile phase B was a 2:1 mixed solvent of mobile phase A and methanol. The green tea leaf extracts were analyzed using a linear gradient from 0–80% B over 0–27 min and 80% B over 27–37 min. The flow rate was 0.5 mL/min, the injection volume was 10 μL and the temperature of the column oven was maintained at 40 °C.

A 2020 quadrupole mass spectrometer (Shimadzu, Kyoto, Japan) equipped with a positive/negative ESI source was used as the detector. The mass spectrometer was operated in negative selected-ion-monitoring (SIM) mode with a capillary voltage of 1.2 V for phenolic compound identification and in positive SIM mode for betanin. The conditions for MS analysis were as follows: spray voltage of −3.5 V, dissolving line temperature of 250 °C, nebulizer gas flow of 1.5 L/min, heat block temperature of 200 °C, drying gas flow rates of 12.00 and 15.00 for phenolics and betanin, respectively and detector voltage of 1.2 V.

### 2.5. DPPH Radical Scavenging Assay

#### 2.5.1. Measurement of DPPH Radical Scavenging Activity Using Green Tea Extract Solution

The radical scavenging activity was determined by the DPPH (1,1-diphenyl-2-picryl-hydrazyl) assay as described previously with some modifications [24]. Prior to adding the DPPH radical, a DMSO solution of 1 μL of the green tea extract solution was added to 0.5 mL of water and incubated at 25, 50, or 90 °C for 5 min. An ethanol solution of the DPPH radical (0.5 mL, 60 μM) was added to the incubated solution and further incubated at 30 °C for 30 min (*n* = 6). The scavenging activity was estimated from OD readings at 515 nm using a microplate reader (Multiskan FC, Thermo Fisher Scientific, Waltham, CA, USA). Samples consisting of 1 μL of DMSO added to 1.0 mL of an ethanol–water solution were also prepared as negative controls. Absorbance readings were converted into percent radical scavenging activity as follows: [(absorbance of the control − absorbance of the sample)/absorbance of the control] × 100.

#### 2.5.2. Measurement of DPPH Radical Scavenging Capacity Using Green Tea Extract–Adsorbed Gelatin

Before the reaction with DPPH, the gelatin to which the green tea extract was adsorbed was washed thrice by adding 0.5 mL of ethanol, the solvent used in the reaction, vortexing and centrifuging to remove the supernatant and then washed thrice with water. The washed gelatin complex was incubated at 25, 50, or 90 °C for 5 min. After incubation, 0.5 mL of water and an ethanol solution of the DPPH radical (60 μM) were added to the incubated solution and vortexed vigorously [25] following which it was further incubated at 30 °C for 30 min (*n* = 6). After the reaction was complete, the mixture was vigorously vortexed and centrifuged at 15,000 rpm for 5 min, following which the absorbance of the supernatant at 515 nm was measured. The radical scavenging activity was calculated in the same manner as described in Section 2.5.1.

#### 2.5.3. Measurement of ABTS Radical Scavenging Activity Using Green Tea Extract

The radical scavenging activity was determined by the ABTS (3-ethylbenzothiazoline-6-sulfonic acid) assay as described previously, with some modifications [26]. First, 5 mL of 7 mM ABTS solution and 88 μL of 140 mM potassium persulfate were mixed and left in the dark at 25 °C for 12 to 16 h. The ABTS solution was diluted with ethanol until it exhibited an absorbance of 0.7 at 740 nm (approximately 50-fold dilution). Prior to the addition of the ABTS radical, a DMSO solution of 1 μL of the green tea extract solution was added to 0.5 mL of water and incubated at 25, 50, or 90 °C for 5 min. Then, 100 μL of the solutions incubated at each temperature and 100 μL of the ABTS solution were mixed and further incubated at 30 °C for 4 min. Afterward and the absorbance was measured at 740 nm (*n* = 8). The scavenging activity was estimated from OD readings at 740 nm using a microplate reader (Multiskan FC, Thermo Fisher Scientific, Waltham, CA, USA). Samples consisting of 1 μL of DMSO added to 0.5 mL of a water solution were also prepared as negative controls. Absorbance was converted into percent radical scavenging activity as follows: [(absorbance of the control − absorbance of the sample)/absorbance of the control] × 100.

#### 2.5.4. Measurement of ABTS Radical Scavenging Activity Using Green Tea Extract–Adsorbed Gelatin

Before the reaction with ABTS, the gelatin to which the green tea extract was adsorbed was washed thrice by adding 0.5 mL of ethanol, the solvent used in the reaction, vortexing and centrifuging to remove the supernatant. It was then washed thrice with water. The washed gelatin complex was incubated at 25, 50, or 90 °C for 5 min. The same amount of ABTS solution as in the suspension of the gelatin complex was added and the mixture was vigorously vortexed [25]. The mixture was incubated at 30 °C for 4 min and the absorbance was measured at 740 nm (*n* = 8). The radical scavenging activity was calculated in the same manner as described in Section 2.5.3.

## 3. Results and Discussion

### 3.1. Adsorption of Flavan-3-ol Derivatives on Gelatin

**Figure 1:**
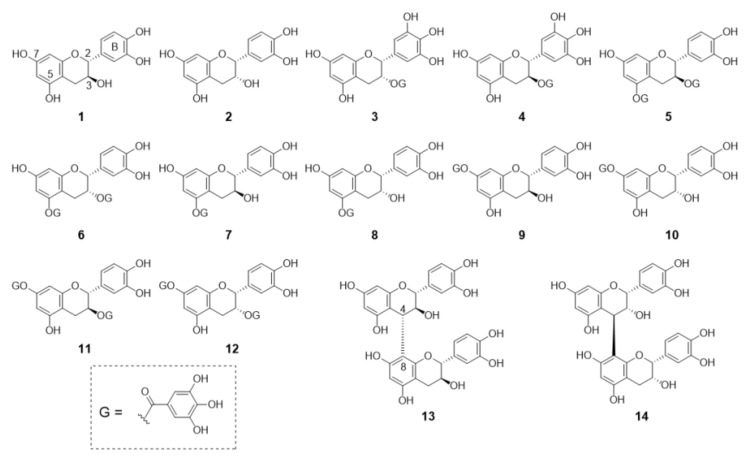
Chemical structures of flavan-3-ol derivatives adsorbed on gelatin.

Figure 1 shows the structures of the flavan-3-ol derivatives used in this study. Compounds **1**–**4** are commercially available and the others were synthesized by us. (+)-Catechin (**1**) and (−)-epicatechin (**2**) do not have a galloyl group, but each has a catechol structure on the B-ring that is considered to interact with gelatin. Given that “G” in the structural formula indicates a galloyl moiety, (−)-epigallocatechin-3-*O*-galate (EGCG) (**3**) and (+)-gallocatechin-3-*O*-galate (GCG) (**4**) have one galloyl moiety at the 3-position and each B-ring has a gallo-structure and, thus, there are considered to be two sites for their interaction with gelatin. (+)-Catechin (**1**)/(−)-epicatechin (**2**) and EGCG (**3**)/GCG (**4**) are isomers with different steric arrangements at the 3-position. Compounds **5**–**12** were all synthesized for SAR research and have been used for selective synthesis to convert the 5- and 7-positions [8,12,13]. Compounds **5**, **7**, **9** and **11** are derived from (+)-catechin (**1**) and compounds **7**, **6**, **8**, **10** and **12** are derived from (−)-epicatechin (**2**). Compounds **5** and **6** have galloyl groups at the 3- and 5-positions and are derived from (+)-catechin (**1**) and (−)-epicatechin (**2**), respectively. Compounds **7** and **8** have a galloyl group only at the 5-position and compounds **9** and **10** have a galloyl group only at the 7-position. In addition, compounds **11** and **12** have galloyl groups at both the 3- and 7-position. Since compounds **5**, **6**, **11** and **12** have two galloyl groups in addition, to the catechol structure of the B-ring, they have three intramolecular sites that can interact with gelatin. On the other hand, compounds **7**, **8**, **9** and **10** have a B-ring catechol structure and one galloyl group and, thus, two intramolecular sites that can interact with gelatin. Compounds **13** and **14**, which we have previously synthesized [3,5], are flavan-3-ol dimers of (+)-catechin (**1**) and (−)-epicatechin called proanthocyanidin B3 and proanthocyanidin B2, respectively. They do not have galloyl groups, but they have four catechol structures that can interact with gelatin.

**Figure 2:**
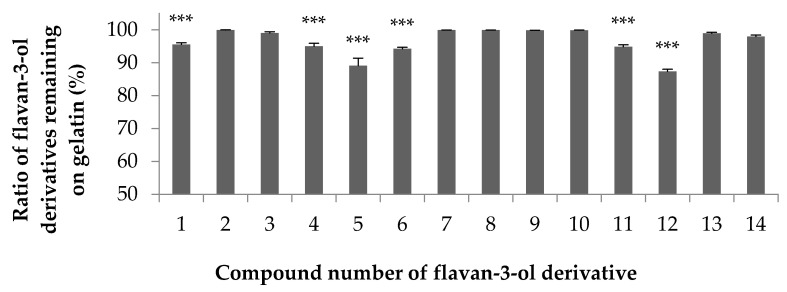
Proportions of flavan-3-ol derivatives **1**–**14** adsorbed on heat-stabilized gelatin after incubation. Error bars represent the SD of the mean (*n* = 6). *** *p* < 0.001 vs. epicatechin (**3**), in Student’s t test. Compounds **2**, **7**, **8**, **9**, **10**, **13** and **14** did not show significantly different data from that of compound **3**.

The interaction between the flavan-3-ol derivatives shown in Figure 1 and gelatin was examined. As the adsorbent, heat-stabilized gelatin that was insoluble in water at room temperature was produced by Nippi Co. and heated at 150 °C for 6 h. Figure 2 shows the ratios of the compounds remaining on gelatin after incubation determined by adding a constant molar concentration of each flavan-3-ol derivative solution to 200 μL of a 10 mg/mL gelatin suspension and incubating. For all the compounds tested, more than 85% was adsorbed on gelatin. In particular, compounds **2**, **3**, **7**–**10**, **13** and **14** did not elute even when almost all the added compound was adsorbed on gelatin and washed. Comparing compounds **1** and **2** suggested that the epicatechin-type structure is slightly more adsorbable. Similar results were obtained for compounds **3** and **4**. In addition, compounds **7**–**10**, with only one galloyl group at the 5- or 7-position, adsorbed more readily on gelatin than compounds **5**, **6**, **11** and **12**, which have two galloyl groups. Interestingly, a greater number of galloyl groups in the molecule does not necessarily cause more binding to gelatin. In addition, the flavan-3-ol dimers **13** and **14** were well adsorbed regardless of their structure. Since compounds **5** to **12** are not commercially available and the only reported examples are trace components of plants, there have been no similar examinations of this interaction.

Hydrophobicity is an important factor in the non-specific interaction between a protein and a small molecule compound and is one of the indicators of whether a small molecule compound can be used as a drug [27]. The water/octanol partition coefficient (Log*P*_OW_), a measure of the hydrophobicity of the compound, was measured to check whether there is any correlation between the interaction shown in Figure 2 and the hydrophobicity of the compound. The results of the shake flask method are listed in Table 1. Compounds **1** to **3** (adsorbed in a relatively large amount) and compounds **5** and **6** (adsorbed in a relatively small amount) were examined. The larger the Log*P*_OW_, the higher the hydrophobicity; conversely, the lower the Log*P*_OW_, the higher the water solubility. As seen in Table 1, because the values of compounds **1** to **3** are low and the values of compounds **5** and **6** are high, compounds **5** and **6** having two moieties of galloyl groups exhibit hydrophobicity even though the number of hydroxyl groups in the molecule is high. From this result, it was suggested that in the flavan-3-ol derivative used in this study, the correlation between the hydrophobicity of the compound and the adsorption may be low. We intend to study the mode of interaction further.

### 3.2. Adsorption of Green Tea Polyphenols on Gelatin

**Figure 3:**
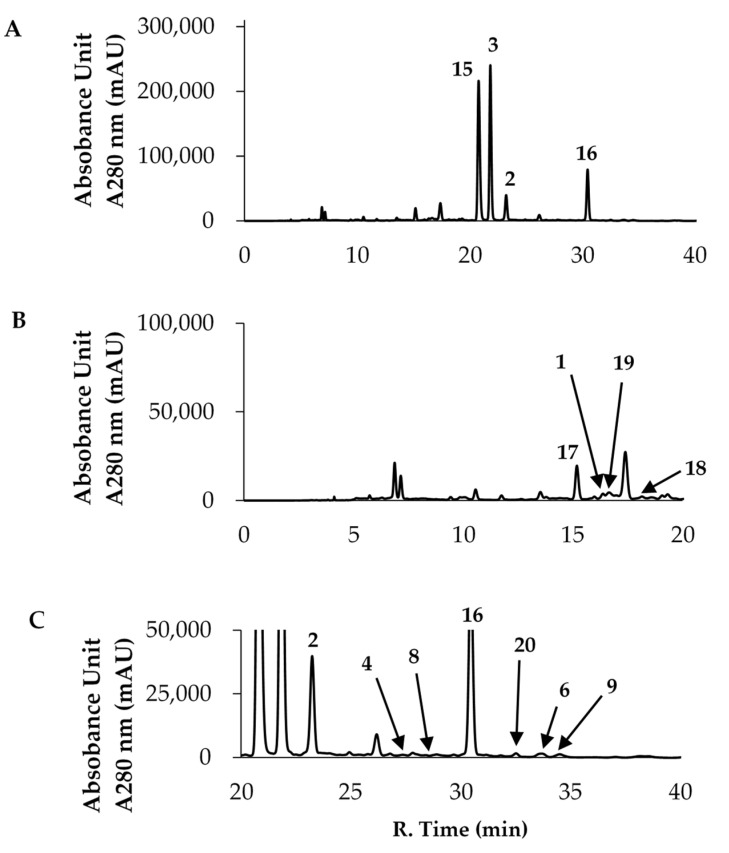
HPLC chromatograms of the methanol extract of green tea leaves. (**A**) Whole chromatogram; (**B**) Magnified chromatogram, 0–20 min; (**C**) Magnified chromatogram, 20–40 min.

**Figure 4:**
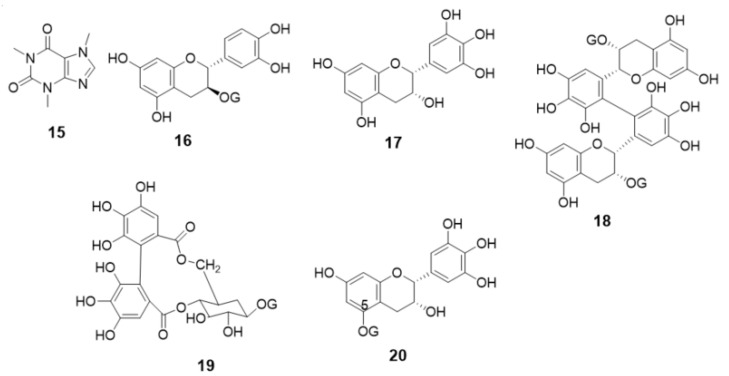
Chemical structures of compounds detected in green tea leaf extract.

It is widely known that green tea is rich in polyphenol compounds. In particular, it contains a large amount of EGCG (**3**), called green tea catechin, which is expected to be responsible for the tea’s functionality [1,2]. The results in Figure 2 confirmed that the flavan-3-ol derivatives, including EGCG (**3**), were adsorbed on heat-stabilized gelatin. Therefore, we decided to use green tea leaves to examine the extent of the adsorption of the contained flavan-3-ol compounds. Figure 3 shows an HPLC chromatogram of the polyphenol mixture from raw green tea leaves extracted with methanol, where A is the entire chromatogram and B and C are magnified portions. The numbers listed in the chromatogram are compound numbers. The green tea leaf extract contained compounds **1**–**4**, **6**, **8** and **9**, as shown in Figure 1, as well as compounds **15**–**20**, as shown in Figure 4.

Figure 3A shows the HPLC chromatogram of the methanol extract of green tea leaves from 0 to 40 min. The major peaks are caffeine (**15**) and EGCG (**3**). In addition, (−)-epicatechin (**2**) and (+)-catechin-3-*O*-gallate (**16**) were present in relatively large amounts. The chromatograms in Figure 3B,C are enlarged versions of 3A. Relatively small amounts of polyphenol components other than the major peaks could also be identified by referring to the LCMS spectral pattern, standard samples and chromatogram pattern reported by Yamamoto et al. [28]. Compound **17** is derived from EGCG (**3**) by hydrolyzation of the galloyl group and compound **18** is a dimer of EGCG (**3**) called Theasinensin A, which is bonded between B-rings. Compound **19** is an ellagitannin called strictinin, with two of its three galloyl groups bound to sugar. Compound **20** is derived from compound **17** by modification of the 5-position with a galloyl group. As described above, the green tea leaf extract contained compounds with various galloyl groups. Considering that these galloyl derivatives can bind to gelatin, we conducted an adsorption experiment on heat-stabilized gelatin.

**Figure 5:**
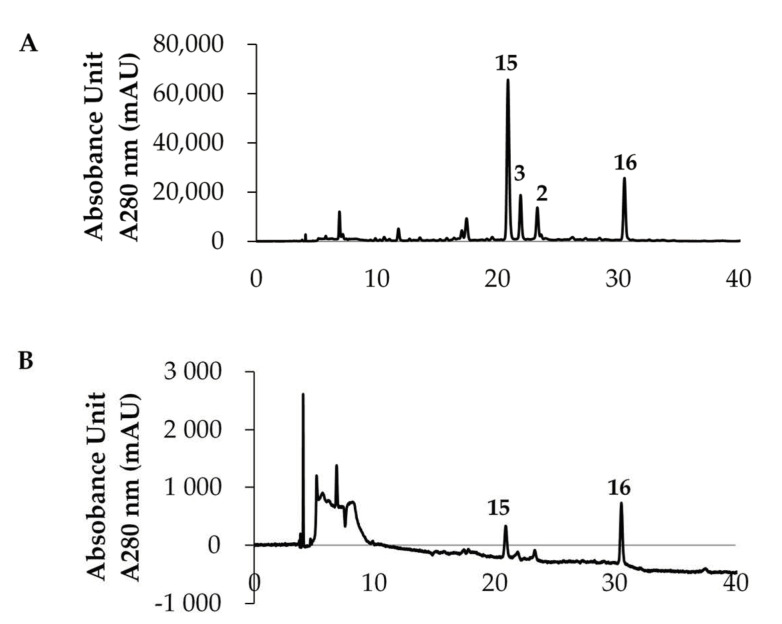
HPLC chromatograms of the supernatant and eluate of gelatin. HPLC chromatogram (**A**) of the supernatant after adding 1 μL of 100 mg/mL green tea leaf extract (equivalent to 100 μg of dried tea leaves) to 100 μL of gelatin suspension (equivalent to 250 μg of dried gelatin) and incubating. HPLC chromatogram of the solution eluted with methanol (**B**).

HPLC chromatograms of the supernatant obtained by adding a DMSO solution (100 mg/mL) of the green tea leaf extract to a 100 μL gelatin suspension, followed by incubation and centrifugation, are shown in Figure 5A. Figure 5B shows an HPLC chromatogram of the compounds eluted from washing the gelatin precipitated in (A) and the green tea leaf extract adsorbed on the surface with methanol. The HPLC analysis confirmed that most of the polyphenol compounds were adsorbed on gelatin; the adsorbed amounts determined by spectrophotometry showed that approximately 97% of the compounds were bound. The peak sizes of caffeine (**15**) and EGCG (**3**) in Figure 5A suggest that caffeine adsorbs less readily on gelatin and is easily recovered (15). The low amount suggests that caffeine (**15**) also adsorbs on gelatin to some extent. Since caffeine (**15**) is a basic compound, it is considered to interact with the acidic amino acids in gelatin. Figure 5B shows that only small amounts of caffeine (**15**) and (+)-catechin-3-*O*-gallate (**16**) were eluted from gelatin upon washing with methanol, whereas most of the other compounds were eluted. Elution was attempted with other solvents and buffer solutions with different pHs, but almost no compounds were eluted from the gelatin. Further, when heated, the gelatin dissolved to form a uniform solution, but the polyphenol compounds did not elute from the gelatin molecules.

### 3.3. DPPH Radical ABTS Radical Scavenging Capacity

The results shown in Figure 3 and Figure 5 suggest that polyphenol compounds were adsorbed on the gelatin treated with green tea extract. Because polyphenol compounds show high antioxidant activities, we aimed to determine whether they exhibited the same activity on gelatin. The DPPH and the ABTS radical scavenging activities of insoluble components of food can be measured even on a solid surface [25].

**Figure 6:**
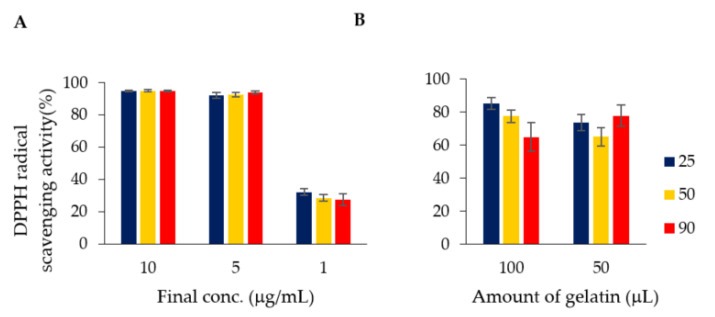
DPPH radical scavenging capacity of green tea extract solution (**A**) and green tea extract–adsorbed gelatin (**B**). The green tea extract (**A**) was measured at final concentrations of 10, 5 and 1 μg/mL. For gelatin (**B**), measurements were performed with 100 and 50 μL of gelatin. RT: Unheated green tea extract or green tea extract–adsorbed gelatin; 50 or 90 °C: Green tea extract or green tea extract–adsorbed gelatin pre-treated at the respective temperature (Blue: 25 °C; Yellow: 50 °C; Red: 90 °C).

The DPPH radical scavenging activity is an indicator of antioxidant activity and has a high correlation with the ability to remove active oxygen species. Similarly, ABTS radical scavenging activity is often used as an index of antioxidant activity of foods and is applicable to a wide range of compounds, ranging from water-soluble to hydrophobic [29]. The antioxidant activity of the flavan-3-ol compound on gelatin was evaluated using these two methods. Figure 6 shows the results of DPPH radical scavenging capacity tests using the green tea leaf extract (A) and the gelatin‑polyphenol complex (B) discussed in the previous section. In addition, to confirm their thermal stability, each sample was heated at 50 or 90 °C for 5 min. As shown in Figure 6B, the compounds adsorbed on gelatin also showed DPPH radical scavenging capacity Although it cannot be directly compared with the results in Figure 6A, where the capacity was measured in solution, it is evident that the capacity depends on the amount of gelatin. As described above, when green tea extract was added to gelatin and incubated, approximately 97% of the compounds were adsorbed. Therefore, it is considered that approximately 50 μg of green tea extract was adsorbed on 50 μL of gelatin. The DPPH radical scavenging capacities, that of the compounds adsorbed on gelatin was lower. However, it is expected that two phenolic hydroxyl groups with a catechol structure are used for adsorption onto gelatin. Because phenolic hydroxyl groups are important in determining the radical scavenging capacity, some reduction in activity is considered appropriate. There was no significant difference in activity with respect to heating. Heating at 90 °C for 5 min was employed with sterilization in mind for use in food, but the activity did not decrease significantly even when adsorbed on gelatin. It was also confirmed by HPLC analysis that the compounds were not released from the gelatin upon heating.

**Figure 7:**
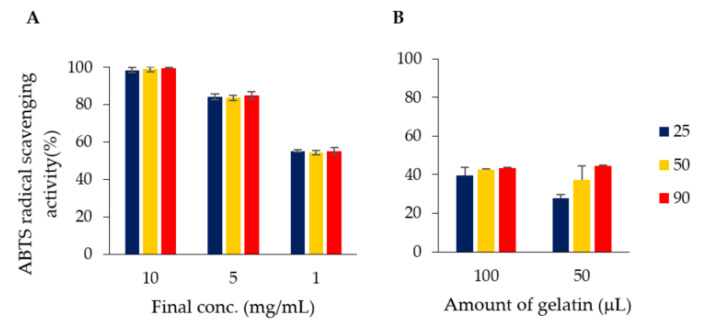
ABTS radical scavenging capacity of green tea extract solution (**A**) and green tea extract–adsorbed gelatin (**B**). The green tea extract (**A**) was measured at final concentrations of 10, 5 and 1 μg/mL. For gelatin (**B**), measurements were performed with 100 and 50 μL of gelatin. RT: Unheated green tea extract or green tea extract–adsorbed gelatin; 50 or 90 °C: Green tea extract or green tea extract–adsorbed gelatin pre-treated at the respective temperature (Blue: 25 °C; Yellow: 50 °C; Red: 90 °C).

Figure 7 shows the results of radical scavenging activity using ABTS radicals. Similar to the DPPH radical scavenging activity, the activity of the gelatin complex tends to decrease; however, it was confirmed that the radical scavenging activity was maintained in the complex.

From these results, it was suggested that polyphenol compounds can be efficiently concentrated using heat-stabilized gelatin and maintain their functionality while adsorbed. Among the polyphenols, compounds with a catechol structure were shown to adsorb well and flavan-3-ol derivatives with higher functionality can be efficiently concentrated. We are currently conducting research to confirm the adsorption of flavan-3-ol derivatives and galloyl molecules on gelatin.

## 4. Conclusions

We confirmed the interaction of heat-stabilized gelatin with flavan-3-ol derivatives, including synthesized compounds. Further, the adsorption of catechol-structured compounds from green tea leaf extract such as the abundant flavan-3-ol derivatives showed that most of the polyphenol compounds could be adsorbed. Because the adsorbed compounds could not be eluted, a DPPH radical and ABTS radical scavenging activity tests were carried out using the as-prepared gelatin–polyphenol complex. Radical scavenging activity was observed while the compounds were adsorbed on gelatin and the activity was not significantly affected by heating at 90 °C for 5 min. These results suggest that functional polyphenols can be efficiently concentrated using heat-stabilized gelatin and maintain their functionality while adsorbed.

## Figures and Tables

**Table 1:** Log*P*_OW_ value calculated from the octanol/water partition coefficient of 1, 2, 3, 5, 6.

Compound No.	Log*P*_OW_
**1**	−0.08
**2**	−0.25
**3**	−0.004
**5**	+0.61
**6**	+1.03

## Data Availability

The data that support the findings of this study are available from the corresponding author, A.S., upon reasonable request.
